# Optimization of School Reintegration for Pediatric Oncology Patients and Their Peers

**DOI:** 10.5334/cie.27

**Published:** 2021-05-17

**Authors:** Savannah Fotheringham, Patrick Karabon, Tracy Wunderlich-Barillas, Janis Traynor, Kate Gowans

**Affiliations:** 1 Oakland University William Beaumont School of Medicine, US; 2 Beaumont Children’s Hospital, US

**Keywords:** pediatric, oncology, peer, school, education

## Abstract

Improved survival rates of pediatric oncology patients give them the opportunity to return to school. This can present a significant challenge, as returning students often become vulnerable to peer rejection. The objective of this double-arm descriptive study was to establish a framework from which to optimize a school reintegration intervention for the peers of pediatric oncology patients. Ultimately, the study aimed to promote increased knowledge, acceptance by peers, and a smooth transition back to school for childhood cancer survivors. We utilized age-appropriate surveys to evaluate the knowledge and concerns of 3^rd^ to 8^th^-grade students in Michigan regarding friends with cancer and to identify concerns of pediatric oncology patients at an academic medical center regarding return to school during or after cancer treatment. The majority of 3^rd^ to 8^th^-grade students correctly answered questions related to etiology, prognosis, side effects, and treatment of cancer. Respondents in 3^rd^ to 5^th^ grade were significantly more likely than 6^th^ to 8^th^ graders to endorse the perception that cancer is contagious (*P* = 0.0036). Fewer students who had a friend with cancer were worried that their friend might die, compared to those who did not have a friend with cancer (3^rd^ to 5^th^ graders [*P* = 0.0002]; 6^th^ to 8^th^ graders [*P* = < 0.0001]). Results suggest that peer intervention may be optimized via customization based upon student concerns rather than focusing on cancer education. Additionally, personalized interventions and assistance for patients should strive to reduce stigma and differentiation from other students.

Though childhood cancer is rare, more than 10,000 children under the age of 15 are diagnosed with cancer in the United States each year (The American Cancer Society [ACS], 2021). Due to advancements in modern medicine, more than 80% of children diagnosed with cancer become long-term survivors (ACS, 2021). The improved survival rates for these children allow them the opportunity to return to school, but that is often a difficult hurdle to overcome given that one in five children with cancer will repeat a class (Barrera et al., 2005), and nearly 50% experience school-related problems following treatment. These children miss an average of 40% of school days during their treatment (af Sandeberg et al., 2008; Charlton et al., 1991; Prevatt et al., 2000), and their school attendance remains irregular up to three years after diagnosis (French et al., 2013; Moore et al., 2009; Prevatt et al., 2000; Suzuki & Kato, 2003).

The nature of a life-threatening disease is stressful and exhausting for patients and their families. Psychological and physical changes make children of any age vulnerable to peer rejection (Fardell et al., 2018; Heffer & Lowe, 2000; Oeffinger et al., 2006; Warner et al., 2016; Yi et al., 2016). Reintegration to a child’s social group is a primary concern for these children, and peer rejection is one of the many challenges they face in returning to school (Butler & Haser, 2006; Gregory et al., 1994). Though challenging, school reintegration for childhood cancer survivors is integral to their academic advancement as well as achievement of normal psychosocial milestones (Fottland, 2000; Heffer & Lowe, 2000; Prevatt et al., 2000; Soejima et al., 2015), and classmate support is a strong predictor of success in these areas (DeLong, 1999; Prevatt et al., 2000).

School reintegration programs at institutions around the United States have attempted to mollify this burden, and there is overwhelming evidence to suggest that these programs assist children in their transition back to school. Such programs typically revolve around several key players: the patient, peers, teachers, parent(s) of both the child and/or their peers, reintegration coordinators/school liaisons, and healthcare providers. School reentry programs increase teachers’ knowledge about the medical and psychosocial aspects of cancer, lead to more positive teacher attitudes toward the child with cancer, and increase teachers’ confidence and comfort levels at managing issues encountered by patients with cancer who are returning to school (Thompson et al., 2015). School reentry programs also increase the knowledge of peers concerning the medical and psychosocial aspects of cancer and improve their attitudes toward and increase their interest in interacting with the student with cancer (Thompson et al., 2015). In addition, they have been perceived by parents, teachers, and the patient to have positive effects (Helms et al., 2016; Katz et al., 1992; Lichtenthal et al., 2016).

Though there is much evidence to support the existence of these programs, there is a notable lack of evidence of their impact on patients or what should comprise the essential elements of school reentry support, including the optimal type and timing of interventions and the necessary expertise or qualifications of personnel implementing the interventions and coordinating support (Lichtenthal et al., 2016; Thompson et al., 2015). Further, these programs as a whole are not standardized across institutions or regions and vary a great deal in what services they offer and who is involved in delivering this assistance.

## The Present Study

Of the many possible avenues to address school reintegration, this dual-armed study focused on patients and their peers. It is the first of its kind to evaluate both patients and peer groups that include 3^rd^ to 8^th^-grade students. Most previous studies in this area have focused on a specific intervention and its impacts on peers’ knowledge of disease facts, willingness to interact with a classmate who has cancer, and attitudes/worries about their classmate with cancer. What they often found was that though knowledge may increase, peers did not change their attitudes or concerns toward a child with cancer, even if they were more willing to interact with them (Benner & Marlow, 1991; Canter & Roberts, 2012; DeLong, 1999; Treiber et al., 1986). Additionally, because the interventions were not standardized and delivery was variable, there are conflicting results quantifying how much the interventions actually impacted the outcomes. Previous studies have also been limited by sample size and the use of convenience samples in one setting (such as a single classroom or school). In addition, there has been more research on adolescent and young adults than elementary and middle school age children (Banko, 1998; Fardell et al., 2018; Pini et al., 2011, 2019; Warner et al., 2016).

The first arm of the study evaluated whether a lack of knowledge of basic cancer topics is potentially impacting the way 3^rd^ to 8^th^-grade students, both those who have had a friend with cancer and those who have not, interact or may interact amid a classmate with cancer. Though previous studies have shown that knowledge may increase through the use of certain interventions, we wanted to establish a baseline from which to determine if lack of knowledge should be addressed and to include a large region rather than measuring the effects on a single classroom or school.

The second arm of the study quantified student worries and concerns, specifically related to cancer patients, that could potentially be addressed in a classroom intervention. Previous studies have demonstrated that these worries and attitudes often do not change with previous interventions, so the present study explored the nature of the concerns in hopes of being able to more specifically address them in the future. Though many studies have looked at the challenges that pediatric oncology patients face going back to school, this arm of the study was designed to explore the patient experience: what things from their viewpoint were difficult, what did they worry about, and what would they want or not want their classmates to know if there were a classroom intervention designed to make their experience better?

## Purpose of Study

The objective of this study was to establish a framework from which to optimize a school reintegration intervention for the peers of pediatric oncology patients that addressed the concerns and possible knowledge gaps of the students and the concerns of the patient, and that integrated the recommendations of patients for such an intervention. The ultimate goal was to aid in the development of an intervention that can be standardized and successful in specific age groups at promoting the growth of knowledge, acceptance by peers, and a smooth transition back to school for childhood cancer survivors. The goal was also to set a precedent for future studies to further evaluate school reintegration and determine the components needed to make successful reintegration possible.

## Methods

### Subjects and Procedures

#### Student Arm

The study included 3^rd^ to 8^th^-grade students (*N* = 186) in Michigan, whose school principal is a member of Michigan Elementary and Middle School Principals Association (MEMSPA). The student arm was carried out via an age-appropriate, nine-question online Qualtrics survey. No existing psychometrically tested questionnaires were available. Two surveys were developed consisting of eight quantitative questions assessing cancer knowledge (etiology, prognosis, treatment, side effects, etc.) and one qualitative free response allowing the respondent to express apprehensions regarding a friend with cancer. Readability for the 3^rd^ to 5^th^- grade survey, assessed by the Flesch-Kincaid Grade Level Test, was at a grade level 3.6. Readability for the 6^th^ to 8^th^-grade survey, assessed by the Flesch-Kincaid Grade Level Test, was at a grade level 4.1.

The student surveys were distributed via email sent from the president of MEMSPA; it is estimated that it reached approximately 1,200 members. MEMSPA members were asked to forward the Qualtrics survey links for each age group as well as a parent information sheet to their school’s 3^rd^ to 8^th^-grade parents/students. By distributing the survey link, parental consent for a child to participate in the study was assumed. Oral assent was assumed for children under the age of 14 years old. Assent from the children 14–17 years old was acquired via an embedded mandatory response consent document in the survey.

#### Patient Arm

This study included patients (*N* = 29) currently receiving cancer therapy or patients who had completed cancer therapy within the past two years at a Michigan academic medical center. The institution did not have an organized school reentry program, but did have a hospital teacher on staff at the time of the study. Patients under the age of eight (<3^rd^ grade) were excluded from the study. Patients over the age of 18 were included if they had been diagnosed before age 18.

The patient arm of the study was carried out via an age-corrected, eight-question online Qualtrics survey. No existing, psychometrically tested questionnaires were available. A survey was developed consisting of five quantitative questions assessing age at diagnosis, school absenteeism, return to school, and worries related to returning to school. The additional three questions were qualitative free response, allowing respondents to describe their transition back to school (if they had returned) and to comment on what they thought should be included or excluded from a school reentry program. Readability for the patient survey, assessed by the Flesch-Kincaid Grade Level Test, was at a grade level 3.8.

The survey was distributed in person during a scheduled clinic visit. Qualified patients/families were given a printed cover letter describing the study as well as a printed information sheet. Patient participants completed the survey online either via a personal electronic device utilizing the paper link or a QR code provided with the cover letter or via a tablet in the office. By distributing the survey link (or allowing survey completion via tablet), parental consent for a child to participate in the study was assumed. Oral assent was assumed for children under the age of 14 years old. Assent from the children 14–17 years old was acquired via an embedded mandatory response assent document in the survey. Consent from the patients 18 years old or older was also acquired via an embedded mandatory response consent document in the survey.

### Data Analysis

The data analysis included both quantitative and qualitative data.

#### Quantitative Analysis

All of the quantitative questions were assessed with answer-choice frequency and percent. All comparisons were analyzed using the chi-square test, of which the assumptions were adequately met. Any *P* value <0.05 (*P* <0.05) indicated a statistically significant result. All analysis for the study was done in SAS 9.4 (SAS Institute Inc.).

While similar, the questions “If you have done something wrong you can get cancer?” in the 3^rd^ to 5^th^-grade survey and “What causes cancer?” in the 6^th^ to 8^th^-grade survey could not be combined due to different response choices. All other questions could be combined into one data set for analysis.

#### Qualitative Analysis

Open-ended questions were reviewed separately by two members of the research team with coding expertise to identify emerging themes. Once coding was complete, disagreements were discussed, and the final results underwent a check for inter-rater reliability using Cohen’s Kappa. The Fisher’s Exact Test was used to compare the qualitative responses of 3^rd^ to 5^th^ graders with 6^th^ to 8^th^ graders. The Fisher’s Exact Test was used because the assumption of adequate expected counts for the chi-square test was not met. Answer-choice frequency and percent were reported.

## Results

The results of this study are presented in the following according to study arm and subsequently quantitative vs. qualitative analysis.

### Student Arm

#### Quantitative Assessment

The majority (>70.00%) of 3^rd^ to 5^th^-grade students (Appendix A1) and 6^th^ to 8^th^-grade students (Appendix A2) answered correctly all of the survey questions related to etiology, prognosis, side effects, and treatment of cancer.

As described in ***Table 1***, 10.09% of the 3^rd^ to 5^th^ graders indicated that cancer is contagious, as opposed to 0% of the 6^th^ to 8^th^ graders (*P* = 0.0036). There was not enough evidence to conclude that any of the other questions were significantly associated with respondents’ grade level (all *P* ≥ 0.05).

**Table 1:** Comparison of Answer-Choice Frequency of 3^rd^ to 5^th^-Grade Students and 6^th^ to 8^th^-Grade Students.


QUESTION	3^RD^ TO 5^TH^ GRADE ANSWER FREQUENCY (%)	6^TH^ TO 8^TH^ GRADE ANSWER FREQUENCY (%)	*P* VALUE

*True or false: If you play with a friend with cancer you can get sick with cancer too. [Cancer is contagious]*			0.0036

True	11 (10.09%)	0 (0.00%)	

False	98 (89.91%)	79 (100.00%)	

*True or false: Cancer can happen to anybody*.			—

True	107 (100.00%)	79 (100.00%)	

False	0 (0.00%)	0 (0.00%)	

*True or false: Everybody who gets cancer will die*.			0.6206

True	5 (4.67%)	5 (6.33%)	

False	102 (95.33%)	74 (93.67%)	

*True or false: Treating cancer is possible but can be hard [difficult]*.			0.3889

True	106 (99.07%)	79 (100.00%)	

False	1 (0.93%)	0 (0.00%)	

*Treating cancer can cause which of these things?*			0.0877

Feeling sick or tired	3 (2.80%)	1 (1.27%)	

Hair loss	13 (12.15%)	3 (3.80%)	

Weight loss or weight gain	1 (0.93%)	0 (0.00%)	

Throwing up	3 (2.80%)	0 (0.00%)	

All of the above	87 (81.31%)	75 (94.94%)	

*What is the best way to treat a friend with cancer?*			0.1090

Never talk about the cancer	3 (2.80%)	6 (7.59%)	

Pretend the cancer is not there and act like nothing is wrong	18 (16.82%)	6 (7.59%)	

Stay away from the friend, you may get cancer too	1 (0.93%)	0 (0.00%)	

Visit and play with them often and ask to help	85 (79.44%)	67 (84.81%)	


A total of 18.69% of 3^rd^ to 5^th^-grade students indicated that they had a friend with cancer compared to 27.85% of 6^th^ to 8^th^-grade students. Having a friend with cancer was not associated with respondents’ grade level (*P* > 0.05, Appendix A3). There was not enough evidence to conclude that any of the quantitative responses of 3^rd^ to 8^th^-grade students were significantly associated with having a friend with cancer (all *P* > 0.05, Appendix A4).

#### Qualitative Assessment

Both the student and the patient arm of the study utilized themes that were confirmed with a Cohen’s Kappa inter-rater reliability analysis, described in ***Table 2***.

**Table 2:** Cohen’s Kappa Inter-Rater Reliability Analysis for Student and Patient Arms.


QUESTION	PERCENT AGREEMENT	KAPPA (95% CI)

Overall Agreement	92.40%	0.91 (0.86, 0.96)

Describe Transition Back to School	85.71%	0.84 (0.72, 0.96)

Describe What Should Be Included in a Program	100.00%	1.00

Describe What Should Not Be Included in a Program	95.24%	0.93 (0.67, 1.19)

3^rd^ to 5^th^ Grade: Worries/Apprehensions About Friend With Cancer; Friend With Cancer	87.50%	0.83 (0.58, 1.00)

3^rd^ to 5^th^ Grade: Worries/Apprehensions About Friend With Cancer; No Friend With Cancer	95.12%	0.94 (0.82, 1.05)

6^th^ to 8^th^ Grade: Worries/Apprehensions About Friend With Cancer; Friend With Cancer	91.30%	0.89 (0.70, 1.08)

6^th^ to 8^th^ Grade: Worries/Apprehensions About Friend With Cancer; No Friend With Cancer	88.68%	0.83 (0.69, 0.97)


Both overall and by question, there was *Almost Perfect Agreement* or *Perfect Agreement* between the two raters for every question. Raters’ scoring sheets were reconciled for the final coding.

The Michigan 3^rd^ to 8^th^-grade student concerns pertaining to a friend with cancer are highlighted in Appendices B1–B4. There was no difference between the qualitative responses of 3^rd^ to 5^th^-grade students and 6^th^ to 8^th^-grade students on whether or not they had a friend with cancer (all *P* > 0.05, Appendices B5–B7).

As illustrated in ***Table 3***, among the 3^rd^ to 5^th^-grade students, fewer students who had a friend with cancer (12.50%) were worried that their friend might die than those who did not have a friend with cancer (55.93%, *P* = 0.0002). There was not enough evidence to determine whether there were any other significant theme differences between these groups (all *P* > 0.05).

**Table 3:** Comparison Between Responses of 3^rd^ to 5^th^ Graders With and Without a Friend With Cancer.


THEME	FRIEND (*N* = 16)	NO FRIEND (*N* = 59)	*P* VALUE

Watching them get sick/suffer	7 (43.75%)	20 (33.90%)	0.4654

Not being able to visit/play	5 (31.25%)	14 (23.73%)	0.5419

Worried they might die	2 (12.50%)	33 (55.93%)	0.0002

Make me feel sad	1 (6.25%)	5 (8.47%)	0.7718

Not knowing how to treat them/respond	1 (6.25%)	2 (3.39%)	0.6031


As illustrated in ***Table 4***, among the 6^th^ to 8^th^-grade students, fewer students who had a friend with cancer (5.26%) were worried that their friend might die than those who did not have a friend with cancer (69.05%, *P* = <0.0001). There was not enough evidence to determine whether here were any other significant theme differences between these older groups (all *P* > 0.05).

**Table 4:** Comparison Between Responses of 6^th^ to 8^th^ Graders With and Without a Friend With Cancer.


THEME	FRIEND (*N* = 19)	NO FRIEND (*N* = 42)	*P* VALUE

Watching them get sick/suffer	7 (36.84%)	10 (23.81%)	0.6031

Not being able to visit/play	6 (31.58%)	5 (11.90%)	0.0643

Worried they might die	1 (5.26%)	29 (69.05%)	<0.0001

Make me feel sad	2 (10.53%)	4 (9.52%)	0.9045

Not knowing how to treat them/respond	1 (5.26%)	1 (2.38%)	0.5552


### Patient Arm

Twenty-nine patient participants responded out of the 32 who received patient surveys (response rate = 90.63%).

#### Quantitative Assessment

An overview of age at diagnosis and when they completed the survey, school absenteeism, and what concerns patient respondents had upon returning to school may be found in Appendix C1.

When comparing age to apprehensions about going back to school (***Table 5***), 70% of patients 11 or younger were worried about their physical appearance going back to school and 20% of patients 12 or older were worried about how they looked going back to school. Respondents who were 11 and younger were significantly more likely to be worried about their appearance going back to school than respondents who were 12 or older (*P* = 0.0124). There was not enough evidence to conclude that any of the other questions were significantly associated with the age of the respondent (all *P* > 0.05).

**Table 5:** Comparison of Age and Frequency of Patient Responses to Reintegration Apprehensions.


QUESTION	11 OR YOUNGER (*N* = 10)	12 OR OLDER (*N* = 15)	*P* VALUE

*Going back to school I was worried about … (select all that apply)*			

Too much attention from teachers and classmates	5 (50.00%)	6 (40.00%)	0.6217

Not enough attention from teachers and classmates	1 (10.00%)	1 (6.67%)	0.7634

How I looked	7 (70.00%)	3 (20.00%)	0.0124

Not being able to catch up with classwork	7 (70.00%)	11 (73.33%)	0.8557

Being treated differently	8 (80.00%)	7 (46.67%)	0.0956

My classmates not understanding	3 (30.00%)	3 (20.00%)	0.5663

Being sick or tired at school	6 (60.00%)	9 (60.00%)	0.9999

I wasn’t worried	1 (10.00%)	2 (13.33%)	0.8016

Other things	0 (0.00%)	0 (0.00%)	—


There was not enough evidence to conclude that any of the multiple-choice concerns were significantly associated with the length of time out of school (all *P* > 0.05) (Appendix C2).

#### Qualitative Assessment

Patient responses when asked to describe their transition back to school, what should be included in a school reentry program, and what should not be included in a school reentry program are described in ***Tables 6, 7*** and ***8***.

**Table 6:** Frequency of Patient Responses Describing Their Transition Back to School.


THEME	FREQUENCY (%) (*N* = 26)

Challenging All Around, But Got Better	2 (7.69%)

School Logistics^a^	2 (7.69%)

Medically Challenging^b^	5 (19.23%)

Academically Challenging^c^	7 (26.92%)

Stigma^d^	4 (15.38%)

Increased Absenteeism	2 (7.69%)

Challenging All Around	1 (3.85%)

Smooth Transition	5 (19.23%)

Teachers Supportive	4 (15.38%)


^a^ Includes meeting with teachers, school coordinating credits/homework. ^b^ Includes side effects from cancer therapy, maintaining focus when ill. ^c^ Includes falling behind in work, difficulty learning lessons, not having enough time to complete work. ^d^ Includes trouble reintegrating with friends, bullying, worry about appearance.

**Table 7:** Frequency of Patient Responses Describing What Should Be Included in a School Reentry Program.


THEME	FREQUENCY (%) (*N* = 29)

Meeting with Teachers	3 (10.34%)

School Coordination	3 (10.34%)

Academic Assistance^a^	10 (34.48%)

Help for Sick Days^b^	1 (3.45%)

Encouragement	1 (3.45%)

Support Groups	6 (20.69%)

Education^c^	6 (20.69%)

Not Sure	3 (10.34%)


^a^ Includes tutoring, one-on-one help, pass/fail, more time for homework. ^b^ Includes rest, time off, back-up plan for parent pick-up. ^c^ Include peer/teacher education about side effects, appearance, and/or communication.

**Table 8:** Frequency of Patient Responses Describing What Should Not Be Included in a School Reentry Program.


THEME	FREQUENCY (%) (*N* = 21)

Differ Assistance With Isolating/Differentiating Others	7 (33.33%)

Physical Activity	1 (4.76%)

Not Sure	10 (47.62%)

Exclude Nothing	1 (4.76%)

No Not Do Nothing	1 (4.76%)

Rush Learning/Assignments	1 (4.76%)


## Discussion

### Student Arm

#### Quantitative Assessment

Though previous studies (Benner & Marlow, 1991; Canter & Roberts, 2012; DeLong, 1999; Treiber et al., 1986) have demonstrated an increase in peer knowledge after educational interventions, our study demonstrated that most students in both 3^rd^ to 5^th^ grade and 6^th^ to 8^th^ grade responded correctly on all survey questions related to cancer etiology, prognosis, treatment, and side effects. This suggests that peer interventions should not exclude, but also not continue to focus on disease facts if students already have a good foundation of knowledge. Instead, interventions should emphasize peer relationships and individual student apprehension. Moreover, previous studies (referenced above) have often confirmed that even with an increase in factual information, students may not change their attitudes towards a classmate with cancer. Thus, this results merely in increased student knowledge with little to no effect on the child the intervention is intended to support. Individual classroom knowledge should be considered before interventions are established. Though most students have a strong foundation of basic cancer facts, not all disease-based facts should be excluded from a peer intervention. As an example, significantly more of the 3^rd^ to 5^th^ graders (10.09%) in this study indicated that they thought cancer was contagious than the 6^th^ to 8^th^ graders (0%, *P* = 0.0036). Thus, younger students may be more likely to broaden the concept of contagion to noncommunicable diseases; however, only the minority of students responded in this way.

#### Qualitative Assessment

The three most frequently reported concerns for 3^rd^ to 5^th^-grade students who have had a friend with cancer included (a) watching them get sick/suffer (43.75%), (b) not being able to visit/play (31.25%), and (c) worry that they might die (12.50%). For 3^rd^ to 5^th^-grade students who had not had a friend with cancer, the three most frequently reported responses included (a) worry that they might die (55.93%), (b) watching them get sick/suffer (33.90%), and (c) not being able to visit/play (23.73%). By comparison, for 6^th^ to 8^th^-grade students who have had a friend with cancer, the three most frequently reported concerns included (a) watching them get sick/suffer (36.85%), (b) not being able to visit/play (31.58%), and (c) outlier responses (26.32%). The top three responses for 6^th^ to 8^th^-grade students who had not had a friend with cancer included (a) worry that they might die (69.05%), (b) watching them get sick/suffer (23.81%), and (c) not being able to visit/play (11.90%). Of these common concerns, fewer 3^rd^ to 8^th^-grade students who had a friend with cancer were worried that their friend might die than those who did not have a friend with cancer (3^rd^ to 5^th^ graders [*P* = 0.0002] and 6^th^ to 8^th^ graders [*P* = < 0.0001]).

These data show that the majority of peers’ concerns are consistent through 3^rd^ to 8^th^-grade students and may allow for standardizing interventions that focus on these concerns for these age groups. However, within each age group, students who had a friend with cancer were significantly less likely to be concerned about their friend dying than those who did not have a friend with cancer. This may be a result of additional education or interaction with their friend and/or their friend’s family easing this concern. These findings suggest, therefore, that when developing an intervention for children who have not previously had a friend with cancer, that prognosis should be addressed with an emphasis on the overall encouraging data that support high survivorship for most childhood cancer patients. Students in the 6^th^ to 8^th^-grade age group with a friend with cancer also seemed to be more concerned about the inability to visit/play than in those who did not have a friend with cancer. Though this was not statistically significant, it may demonstrate that interaction with their friend and/or their friend’s family may diminish some concerns but increase others, especially within this age range where socialization and peer connection are highly valued.

### Patient Arm

#### Quantitative Assessment

All patient respondents (100%) had returned to school since beginning treatment at the time they completed the survey. The mean age of diagnosis for these respondents was 11.63 years old. At completion of the survey, 30.95% of respondent were 18 years old or older, 38.10% were 14–17 years old, and 30.95% were <14 years old. The majority had missed between 2–6 months (38.71%) and 6–12 months (22.58%) of school, confirming the existence of a significant amount of absenteeism in the pediatric cancer population.

The top three worries that patients experienced included (a) not being able to catch up with classwork (65.52%), (b) feeling sick or tired at school (58.62%), and (c) being treated differently (51.72%). A significant number of respondents also acknowledged other worries, including receiving insufficient or too much attention from teachers and classmates, how they looked, and their classmates not understanding their situation. Of these respondents, 10.34% acknowledged not having worries. Respondents who were 11 and younger were significantly more likely to be worried about their appearance (70.00%) when going back to school than respondents who were 12 or older (20.00%, *P* = 0.0124). This discrepancy may be another reason to consider including education about side effects to peers in the younger age groups. It also is an indication of the need to ensure younger patients have a support system and/or counseling in place in the event that they face bullying and/or psychosocial consequences of their anxieties in this area.

#### Qualitative Assessment

When asked to describe their transition back to school, the two most frequently responses by patients indicated that it was both academically (26.92%) and medically (19.23%) challenging. At the same time, 19.23% acknowledged that they had a smooth transition back to school and 15.38% acknowledged that their teachers were supportive. Other patients noted that stigma played a significant role in their transition (15.38%), transitioning overall was challenging but got better (7.69%), the logistics with the school were difficult (7.69%), absences were difficult (7.69%), and that it was overall challenging (3.85%). For example, many patients mentioned how it was medically challenging to return to school, “Some days I got to school and within an hour I’d feel sick from chemo, and it was very difficult to focus. Some days I felt like I was going to throw up, and I just wanted to leave and go home so that it wouldn’t happen in the middle of class.” These responses are verifying evidence of the many challenges this population faces when returning to school.

When asked to describe what should be included in a program, patients’ top three responses included (a) academic assistance (34.48%), (b) support groups (20.69%), and (c) education (20.69%). For example, many patients wished that they had received more academic assistance before returning to the classroom, “[Include] a way for the students to meet their teachers prior to their return to school so they can get to know one another and devise a plan for the school year” and once back in school, “Help with work missed and time to get back energy.” Some patients noted a desire for support groups, “I would like to be able to communicate with other teens my age that have gone through this. We could help each other and transition together.” These responses indicate the need for future robust school reentry programs to include both peer intervention and coordination with school and teachers to provide a safe, healthy, accommodating, and stimulating environment for these patients to continue to learn and grow. Additionally, hospitals and healthcare teams should consider incorporating support groups for patients in the same age groups to facilitate encouragement from each other as they transition back to school together.

When asked to describe what should not be included in a program, patients’ top response was not to isolate or differentiate them from others when giving assistance (33.33%). Many patients were unsure what should be excluded (47.62%). The most prominent response was an emphasis on the desire to limit the differentiation from other students while still giving them help, “[Exclude] anything that would make other students feel like they weren’t getting treated fairly compared to the child with the sickness. I never liked the attention because I wanted to be treated like every other student.” While cancer patients who return to school overwhelmingly desire assistance, they do not want to be set apart from their peers in a way that gives them noticeable special treatment or labels them as different. This was also evident, though not statistically significant, in the multiple-choice question about reintegration concerns.

## Conclusion

The majority of 3^rd^ to 8^th^-grade students correctly answered questions related to etiology, prognosis, side effects, and treatment of cancer although 3^rd^ to 5^th^ graders were significantly more likely than 6^th^ to 8^th^ graders to endorse the impression that cancer is contagious. While there were multiple common concerns between the two student age groups, significantly fewer 3^rd^ to 8^th^ graders who had a friend with cancer were worried that their friend might die than those who did not. This finding suggests that intervention should be customized to specific student and class concerns rather than focusing solely on cancer education.

Though the patient participants reported a wide array of concerns regarding return to school, patients who were 11 and younger were significantly more likely to be worried about their appearance going back to school than respondents who were 12 or older. When asked to describe what should not be included in a program, patients’ top response was not to single them out from their classmates, which suggests that interventions and assistance for patients should aim to reduce stigma and differentiation from other students. Additionally, programs designed to help children with cancer return to school successfully should include discrete academic assistance as well as disease education for younger children. Academic support should be considered part of a holistic approach to ensuring positive outcomes for this population. Institutions that provide care to pediatric oncology patients should consider incorporating this type of customized school reentry support as a new standard of care.

### Strengths and Limitations

This study is the first of its kind to evaluate and integrate the concerns of both pediatric oncology patients and their peers as the patients face the intimidating prospect of returning to school either during or following cancer treatment. Instead of using a workshop or presentation to guide research and future intervention, this study used a needs assessment approach to collect information to guide future interventions. The study also included a broader student sample size and distribution than previous studies related to this topic.

The surveys used in this study were not first tested on a pilot sample of members of the target population. Consequently, we were not able to identify whether respondents completely understood the questions and instructions, and whether the meaning of questions was the same for all respondents. For these reasons, we were not able to determine whether sufficient response categories were available for the closed-ended questions. However, there were multiple options for free response. We could not compare one question on the student survey because of a survey design that limited our data for that specific question. We also did not know which students received parent assistance in the completion of surveys.

Patient data were only collected at a single academic medical center, which may not be generalizable to the entire pediatric oncology population. Further, we did not collect respondent descriptors such as race, gender, or zip code for either the student or patient populations. These differences could impact the results of this study.

Though the student surveys were sent via MEMSPA with an endorsement from the president of MEMSPA (cover letter and in-person address at the association’s annual conference), the response rate was lower than expected, perhaps underscoring the need to remove a stigma that is still looming over children and adolescents with a cancer history who need their career as a student to remain an important part of their identity. The methodology of survey distribution made it difficult to obtain an actual response rate due to the inability to predict how many principals actually forwarded the surveys and to how many parents/students. In addition, performing the research at a small oncology program contributed to a smaller patient sample size.

### Further Research

Further research on how to address student concerns when their classmate has been treated or is being treated for cancer is essential. It is obvious through this and past research that education on disease information may not be as helpful in producing a positive outcome for the patient as focusing on peer worries and apprehensions. The consistent nature of the peers’ concern in this study may enable an intervention that is suitable for all 3^rd^ to 8^th^-grade students. Additionally, future interventions should involve the patients’ peers and school faculty in a way that does not isolate, differentiate, or set apart the patient in a negative manner.

The study sets a precedent for future studies to evaluate specific aspects of school reintegration such as academic assistance (both physical education and classroom education), parent/family assistance, teacher and school coordination, and coordination with the healthcare team. Given the implicit level of heterogeneity in school systems across the country, such studies could help to determine the necessary components of successful school reintegration and the degree to which standardization in this realm is feasible.

## Additional Files

The additional files for this article can be found as follows:

10.5334/cie.27.s1 Appendix A. 3^rd^ to 8^th^-grade student quantitative analysis.

10.5334/cie.27.s2 Appendix B. 3^rd^ to 8^th^-grade student qualitative analysis.

10.5334/cie.27.s3 Appendix C. Patient quantitative analysis.

10.5334/cie.27.s4 Appendix D. 3^rd^ to 8^th^-grade student and patient surveys.
