# Supplementary material for: Optimization of School Reintegration for Pediatric Oncology Patients and Their Peers
Source: Contin Educ. 2021 May 17;2(1):60–72. doi: 10.5334/cie.27 (PMC11104304; doi:10.5334/cie.27)
Supplement: Appendix A. — 3rd to 8th-grade student quantitative analysis. [file cie-2-1-27-s1.pdf]

## Supplementary Files: Appendices

## Appendix A:

| Question | Answer Frequency (Percent) |
|---|---|
| *True or false: If you play with a friend with cancer you can get sick with cancer too. (n = 109)* | |
| True | 11 (10.09%) |
| False | 98 (89.91%) |
| *True or false: If you have done something wrong you can get cancer. (n = 107)* | |
| True | 12 (11.21%) |
| False | 95 (88.79%) |
| *True or false: Cancer can happen to anybody. (n = 107)* | |
| True | 107 (100.00%) |
| False | 0 (0.00%) |
| *True or false: Everybody who gets cancer will die. (n = 107)* | |
| True | 5 (4.67%) |
| False | 102 (95.33%) |
| *True or false: Treating cancer is possible but can be hard. (n = 107)* | |
| True | 106 (99.07%) |
| False | 1 (0.93%) |
| *Treating cancer can cause which of these things? (n = 107)* | |
| Feeling sick or tired | 3 (2.80%) |
| Hair loss | 13 (12.15%) |
| Weight loss or weight gain | 1 (0.93%) |
| Throwing up | 3 (2.80%) |
| All of the above | 87 (81.31%) |
| *What is the best way to treat a friend with cancer? (n = 107)* | |
| Never talk about the cancer | 3 (2.80%) |
| Pretend the cancer is not there and act like nothing is wrong | 18 (16.82%) |
| Stay away from the friend, you may get cancer too | 1 (0.93%) |
| Visit and play with them often and ask to help | 85 (79.44%) |

**A1:** 3$^{rd}$-5$^{th}$ grade student responses to survey questions related to etiology, prognosis, side effects, and treatment of cancer.

| Question | Answer Frequency (Percent) |
|---|---|
| *True or false: Cancer is contagious. (n = 79)* | |
| True | 0 (0.00%) |
| False | 79 (100.00%) |
| *What causes cancer? (n = 79)* | |
| It is in your genes | 6 (7.59%) |
| Contracted from somebody else who has cancer | 0 (0.00%) |
| Environment or lifestyle choices (ex: smoking…) | 17 (21.52%) |
| Older age | 0 (0.00%) |
| A, C, and D | 56 (70.89%) |
| *True or false: Cancer can happen to anybody. (n = 79)* | |
| True | 79 (100.00%) |
| False | 0 (0.00%) |
| *True or false: Everybody who gets cancer will die. (n = 79)* | |
| True | 5 (6.33%) |
| False | 74 (93.67%) |
| *True or false: Treating cancer is possible but sometimes difficult. (n = 79)* | |
| True | 79 (100.00%) |
| False | 0 (0.00%) |
| *Treating cancer can cause which of these things? (n = 79)* | |
| Feeling sick or tired | 1 (1.27%) |
| Hair loss | 3 (3.80%) |
| Weight loss or weight gain | 0 (0.00%) |
| Throwing up | 0 (0.00%) |
| All of the above | 75 (94.94%) |
| *What is the best way to treat a friend with cancer? (n = 79)* | |
| Never talk about the cancer | 6 (7.59%) |
| Pretend the cancer is not there and act like nothing is wrong | 6 (7.59%) |
| Avoid the friend, you may get cancer too | 0 (0.00%) |

| | |
|---|---|
| Visit them often and ask if they need help with anything | 67 (84.81%) |

**A2:** 6$^{th}$-8$^{th}$ grade student responses to survey questions related to etiology, prognosis, side effects, and treatment of cancer.

| Question | 3$^{rd}$-5$^{th}$ Grade Answer Frequency (%) | 6$^{th}$-8$^{th}$ Grade Answer Frequency (%) | P-Value |
|---|---|---|---|
| *Have you had a friend with cancer?* | *(n=107)* | *(n=79)* | 0.1399 |
| Yes | 20 (18.69%) | 22 (27.85%) | |
| No | 87 (81.31%) | 57 (72.15%) | |

**A3:** Frequency of respondents indicating they had or did not have a friend with cancer. Association of having or not having a friend with cancer with grade level.

| Question | Yes (n=42) | No (n=144) | P-Value |
|---|---|---|---|
| *True or false: If you play with a friend with cancer you can get sick with cancer too. [Cancer is contagious]* | | | 0.0726 |
| True | 0 (0.00%) | 11 (7.64%) | |
| False | 42 (100.00%) | 133 (92.36%) | |
| *True or false: Cancer can happen to anybody.* | | | --- |
| True | 42 (100.00%) | 144 (100.00%) | |
| False | 0 (0.00%) | 0 (0.00%) | |
| *True or false: Everybody who gets cancer will die.* | | | 0.3280 |
| True | 1 (2.38%) | 9 (6.25%) | |
| False | 41 (97.62%) | 135 (93.75%) | |
| *True or false: Treating cancer is possible but can be hard [difficult].* | | | 0.5881 |
| True | 42 (100.00%) | 143 (99.31%) | |
| False | 0 (0.00%) | 1 (0.69%) | |
| *Treating cancer can cause which of these things?* | | | 0.2429 |
| Feeling sick or tired | 0 (0.00%) | 4 (2.78%) | |
| Hair loss | 1 (2.38%) | 15 (10.42%) | |
| Weight loss or weight gain | 0 (0.00%) | 1 (0.69%) | |
| Throwing up | 0 (0.00%) | 3 (2.08%) | |
| All of the above | 41 (97.62%) | 121 (84.03%) | |
| *What is the best way to treat a friend with cancer?* | | | 0.9501 |
| Never talk about the cancer | 2 (4.76%) | 7 (4.86%) | |
| Pretend the cancer is not there and act like nothing is wrong | 5 (11.90%) | 19 (13.19%) | |
| Stay away from the friend, you may get cancer too | 0 (0.00%) | 1 (0.69%) | |
| Visit and play with them often and ask to help | 35 (83.33%) | 117 (81.25%) | |

**A4:** Response comparison of respondents 3$^{rd}$-8$^{th}$ grade that indicated they did have a friend with cancer to those who did not.
