# Supplementary material for: Optimization of School Reintegration for Pediatric Oncology Patients and Their Peers
Source: Contin Educ. 2021 May 17;2(1):60–72. doi: 10.5334/cie.27 (PMC11104304; doi:10.5334/cie.27)
Supplement: Appendix B. — 3rd to 8th-grade student qualitative analysis. [file cie-2-1-27-s2.pdf]

Appendix B:

Theme	Frequency (%) (n = 16)
Watching them get sick/suffer	7 (43.75%)
Not being able to visit/play	5 (31.25%)
Not being able to help	1 (6.25%)
I don't remember	1 (6.25%)
Make me feel sad	1 (6.25%)
Worried they might die	2 (12.50%)
Not knowing how to treat them/respond	1 (6.25%)

B1: Michigan 3rd-5th grade students concerns pertaining to a friend with cancer: 3rd-5th grade respondents who indicated that they did have a friend with cancer responded with these themes.

Theme	Frequency (%) (n = 59)
Watching them get sick/suffer	20 (33.90%)
Not being able to visit/play	14 (23.73%)
Not being able to help	1 (1.69%)
Worried that they would die	33 (55.93%)
Not knowing how to treat them/respond	2 (3.39%)
Worried about stigma	7 (11.86%)
Worried about contagious disease	1 (1.69%)
Make me feel sad	5 (8.47%)
Outlier	2 (3.39%)

B2: Michigan 3rd-5th grade students concerns pertaining to a friend with cancer: 3rd-5th grade respondents who indicated that they did not have a friend with cancer responded with these themes.

Theme	Frequency (%) (n = 19)
Watching them get sick/suffer	7 (36.85%)
Not being able to visit/play	6 (31.58%)
Worried that they would die	1 (5.26%)
Worried about stigma	1 (5.26%)
Make me feel sad	2 (10.53%)
Not knowing how to treat them/respond	1 (5.26%)
Not being able to help	2 (10.53%)
Outlier	5 (26.32%)

B3: Michigan 6rd-8th grade students concerns pertaining to a friend with cancer: 6rd-8th grade respondents who indicated that they did have a friend with cancer responded with these themes.

Theme	Frequency (%) (n = 42)
Watching them get sick/suffer	10 (23.81%)
Not being able to visit/play	5 (11.90%)
Worried that they would die	29 (69.05%)
Not knowing how to treat them/respond	1 (2.38%)
Worried about stigma	4 (9.52%)
Make me feel sad	4 (9.52%)
Worried about how friends and family cope	1 (2.38%)
Outlier	1 (2.38%)

B4: Michigan 6rd-8th grade students concerns pertaining to a friend with cancer: 6rd-8th grade respondents who indicated that they did not have a friend with cancer responded with these themes.

Theme	Grade 3rd-5th (n = 16)	Grade 6-8 (n = 19)	P-Value
Watching them get sick/suffer	7 (43.75%)	7 (36.84%)	0.7391
Not being able to visit/play	5 (31.25%)	6 (31.58%)	0.9833
Not being able to help	1 (6.25%)	2 (10.53%)	0.6526
Make me feel sad	1 (6.25%)	2 (10.53%)	0.6526
Worried they might die	2 (12.50%)	1 (5.26%)	0.5820
Not knowing how to treat them/respond	1 (6.25%)	1 (5.26%)	0.9003

B5: Comparison of concerns between 3rd-5th graders and 6th-8th graders who have a friend with cancer.

Theme	Grade 3rd-5th (n = 59)	Grade 6-8 (n = 42)	P-Value
Watching them get sick/suffer	20 (33.90%)	10 (23.81%)	0.3772
Not being able to visit/play	14 (23.73%)	5 (11.90%)	0.1965
Worried they might die	33 (55.93%)	29 (69.05%)	0.2166
Not knowing how to treat them/respond	2 (3.39%)	1 (2.38%)	0.7685
Worried about stigma	7 (11.86%)	4 (9.52%)	0.7588

Make me feel sad	5 (8.47%)	4 (9.52%)	0.8553

B6: Comparison of concerns between 3rd-5th graders and 6th-8th graders who do not have a friend with cancer.

Theme	Grade 3rd-5th (n = 75)	Grade 6th-8th (n = 61)	P-Value
Watching them get sick/suffer	27 (36.00%)	17 (27.87%)	0.3595
Not being able to visit/play	19 (25.33%)	11 (18.03%)	0.4061
Make me feel sad	6 (8.00%)	6 (9.84%)	0.7672
Worried they might die	35 (46.67%)	30 (49.18%)	0.8633
Not knowing how to treat them/respond	3 (4.00%)	2 (3.28%)	0.8241

B7: Comparison of concerns between 3rd-5th graders and 6th-8th graders unrelated to a friend with cancer.
