# Supplementary material for: Optimization of School Reintegration for Pediatric Oncology Patients and Their Peers
Source: Contin Educ. 2021 May 17;2(1):60–72. doi: 10.5334/cie.27 (PMC11104304; doi:10.5334/cie.27)
Supplement: Appendix C. — Patient quantitative analysis. [file cie-2-1-27-s3.pdf]

Appendix C:

At what age were you diagnosed with cancer? (n = 27)	
Mean (Standard Deviation)	11.63 (3.77)
Median (Interquartile Range)	13.00 (8.00, 15.00)
Minimum Value, Maximum Value	4.00, 17.00
How old are you? (n = 42)	
18 years or older	13 (30.95%)
14-17 years old	16 (38.10%)
<14 years old	13 (30.95%)
How much school did you miss because of your illness or treatment? (n = 31)	
<60 days	4 (12.90%)
2-6 months	12 (38.71%)
6-12 months	7 (22.58%)
> 1 year	5 (16.13%)
I can't remember	3 (9.68%)
Have you returned to school since beginning treatment? (n = 31)	
Yes	31 (100.00%)
No	0 (0.00%)
Going back to school I was worried about ... (n = 29) (Check all that apply)	
Too much attention from teachers and classmates	12 (41.38%)
Not enough attention from teachers and classmates	2 (6.90%)
How I looked	11 (37.93%)
Not being able to catch up with classwork	19 (65.52%)
Being Treated Differently	15 (51.72%)
My classmates not understanding	7 (24.14%)
Being sick or tired at school	17 (58.62%)
I wasn't worried	3 (10.34%)
Other things	0 (0.00%)

C1: The age at which patient respondents were diagnosed with cancer, the age when they completed the survey, absenteeism, whether or not they had returned to school since beginning treatment for cancer, and what worries/apprehensions they had upon returning to school.

**Question**	**<6 months (n = 16)**	**>6 months or currently out of school (n = 10)**	**P-Value**
Going back to school I was worried about ... (select all that apply)			
Too much attention from teachers and classmates	5 (31.25%)	6 (60.00%)	0.1489
Not enough attention from teachers and classmates	0 (0.00%)	2 (20.00%)	0.1385
How I looked	5 (31.25%)	5 (50.00%)	0.3390
Not being able to catch up with classwork	11 (68.75%)	7 (70.00%)	0.9464
Being Treated Differently	6 (37.50%)	7 (70.00%)	0.1069
My classmates not understanding	3 (18.75%)	4 (40.00%)	0.2347
Being sick or tired at school	10 (62.50%)	6 (60.00%)	0.8986
I wasn't worried	1 (6.25%)	1 (10.00%)	0.7270
Other things	0 (0.00%)	0 (0.00%)	---

C2: Comparison of length of time out of school to apprehensions about going back to school.
