# Supplementary material for: Optimization of School Reintegration for Pediatric Oncology Patients and Their Peers
Source: Contin Educ. 2021 May 17;2(1):60–72. doi: 10.5334/cie.27 (PMC11104304; doi:10.5334/cie.27)
Supplement: Appendix D. — 3rd to 8th-grade student and patient surveys. [file cie-2-1-27-s4.pdf]

Appendix D:

*All surveys were distributed with corresponding electronic or paper information sheets and assent/consent documentation.

Questions
1. True or false: If you have done something wrong you can get cancer.
2. True or false: If you play with a friend with cancer you can get sick with cancer too.
3. True or false: Cancer can happen to anybody.
4. True or false: Everybody who gets cancer will die.
5. True or false: Treating cancer is possible but can be hard.
6. Treating cancer can cause which of these things?
**a.** Feeling sick or tired
**b.** Hair loss
**c.** Weight loss or weight gain
**d.** Throwing up
**e.** All of the above
7. What is the best way to treat a friend with cancer?
**a.** Never talk about the cancer
**b.** Pretend the cancer is not there and act like nothing is wrong
**c.** Stay away from the friend, you may get cancer too
**d.** Visit and play with them often and ask to help
8. Have you had a friend with cancer?
**a.** Yes
**b.** No
9a. If yes: What things were hard for you as their friend when they were sick?
9b. If no: If you had a friend with cancer, what things might make you feel worried or sad?

D1: Student survey for 3rd-5th grade.

Questions
1. True or false: Cancer is contagious.
2. What causes cancer?
**c.** It is in your genes
**d.** Contracted from somebody else who has cancer
**e.** Environment or lifestyle choices (ex: smoking…)
**f.** Older age
**g.** A, C, and D
3. True or false: Cancer can happen to anybody.
4. True or false: Everybody who gets cancer will die.
5. True or false: Treating cancer is possible but sometimes difficult.
6. Treating cancer can cause which of these things?
**a.** Feeling sick or tired
**b.** Hair loss
**c.** Weight loss or weight gain
**d.** Throwing up
**e.** All of the above
7. What is the best way to treat a friend with cancer?
**a.** Never talk about the cancer
**b.** Pretend the cancer is not there and act like nothing is wrong
**c.** Avoid the friend, you may get cancer too
**d.** Visit them often and ask if they need help with anything
8. Have you had a friend with cancer?
**a.** Yes
**b.** No
9a. If yes: What things were difficult for you as their friend when they were sick?
9b. If no: If you had a friend with cancer, what things might make you feel worried or sad?

D2: Student survey for 6rd-8th grade.

Questions
1. How old are you?
**a.** 18 years or older
**b.** 14-17 years old
**c.** < 14 years old
2. At what age were you diagnosed with cancer?
3. How much school did you miss because of your illness or treatment?
a. < 60 days
b. 2-6 months
c. 6-12 months

	d.	> 1 year
	e.	I can’t remember
4.		***Have you returned to school since beginning treatment?***
	a.	Yes
	b.	No
5a.		***If yes: Describe your transition back to school.***
5a.		***If no: Do you think going back to school will be challenging? Why or why not?***
6a.		***If yes: Mark all that apply concerning going back to school: I was worried about ...***
	**a.**	Too much attention from teachers and classmates
	**b.**	Not enough attention from teachers and classmates
	**c.**	How I looked
	**d.**	Not being able to catch up with classwork
	**e.**	Being treated differently
	**f.**	My classmates not understanding
	**g.**	Being sick or tired at school
	**h.**	I wasn’t worried
	i.	Other- Please specify:
6b.		***If no: Mark all that apply concerning going back to school: I worry about…***
	**j.**	Too much attention from teachers and classmates
	**k.**	Not enough attention from teachers and classmates
	**l.**	How I looked
	**m.**	Not being able to catch up with classwork
	**n.**	Being treated differently
	**o.**	My classmates not understanding
	**p.**	Being sick or tired at school
	**q.**	I am not worried
	r.	Other- Please specify:
7a.		***If yes: If we created a program to help kids like you return to school what things would you like to see happen?***
7b.		***If no: If we created a program to help kids like you return to school what things would you like to see happen?***
8a.		***If yes: What things would you rather not be included into one of these programs?***
8b.		***If no: What things would you rather not be included into one of these programs?***

D3: Patient survey.
